# Altered Cerebellar Circuitry following Thoracic Spinal Cord Injury in Adult Rats

**DOI:** 10.1155/2016/8181393

**Published:** 2016-07-18

**Authors:** Nishant P. Visavadiya, Joe E. Springer

**Affiliations:** ^1^Department of Biomedical Sciences, Quillen College of Medicine, East Tennessee State University, Johnson City, TN 37614, USA; ^2^Department of Physical Medicine and Rehabilitation, Spinal Cord and Brain Injury Research Center, Department of Anatomy and Neurobiology, University of Kentucky, Lexington, KY 40536, USA

## Abstract

Cerebellar function is critical for coordinating movement and motor learning. However, events occurring in the cerebellum following spinal cord injury (SCI) have not been investigated in detail. We provide evidence of SCI-induced cerebellar synaptic changes involving a loss of granule cell parallel fiber input to distal regions of the Purkinje cell dendritic tree. This is accompanied by an apparent increase in synaptic contacts to Purkinje cell proximal dendrites, presumably from climbing fibers originating in the inferior olive. We also observed an early stage injury-induced decrease in the levels of cerebellin-1, a synaptic organizing molecule that is critical for establishing and maintaining parallel fiber-Purkinje cell synaptic integrity. Interestingly, this transsynaptic reorganizational pattern is consistent with that reported during development and in certain transgenic mouse models. To our knowledge, such a reorganizational event has not been described in response to SCI in adult rats. Regardless, the novel results of this study are important for understanding SCI-induced synaptic changes in the cerebellum, which may prove critical for strategies focusing on promoting functional recovery.

## 1. Introduction

Traumatic spinal cord injury (SCI) damages intrinsic local circuits, descending supraspinal pathways involved in motor function, autonomic systems, and ascending fiber systems conveying sensory information and nonconscious proprioception. To date, there are no effective treatments available that reduce these deficits or promote recovery of function. The lack of successful interventions is based, in part, on an incomplete understanding of the biochemical, physiological, and neuroanatomical changes that occur at different time points following injury. The majority of current research in the field of experimental SCI focuses on preservation, restoration, and activation of residual descending motor systems as well as intact intraspinal local circuits to promote recovery of motor function [1]. Several studies in complete spinalized rats and cats have demonstrated remarkable success in promoting body-weight supported and full weight bearing treadmill stepping by manipulating intraspinal circuits [2–7]. However, supraspinal inputs also contribute to the initiation and execution of voluntary nonreflexive overground locomotion [8–10]. Cerebellar function plays an essential role in processing nonconscious proprioceptive information required for ongoing voluntary movement. Therefore, as strategies are developed to promote and restore voluntary movement following spinal cord injury (SCI), it will be important to have a better understanding of potential injury-related changes in cerebellar circuitry that impact recovery of motor function.

In rats, Clarke's nucleus relays nonconscious proprioception from spinal levels T1-L2 via the dorsal spinocerebellar tract (dSCT) to cerebellar lobules I, II, III, and VIII [11, 12]. Axons from neurons in Clarke's nucleus ascend in the dSCT and enter the cerebellum as mossy fibers to innervate cerebellar granule cells. The granule cells then give rise to parallel fibers that provide excitatory afferents to the distal segments of Purkinje cell dendrites [13]. The output of the Purkinje cells to deep cerebellar nuclei is essential for coordinating ongoing voluntary movement. SCI at thoracic levels most often results in the loss of dSCT input to the cerebellum, including nonconscious proprioceptive information conveyed by Clarke's nucleus. In this initial study, we investigated changes in cerebellar circuitry in response to midthoracic SCI and suggest that understanding these changes provides additional insight into altered function of supraspinal pathways following SCI.

## 2. Methods

### 2.1. Spinal Cord Injury and Tissue Processing

A total of 20 adult female Long-Evans rats (Harlan, Indianapolis, IN) weighing 225–250 gm at the time of surgery were used in this study. Animals were maintained under environmentally controlled conditions and subjected to a 12 h/12 h light/dark cycle with food and water provided ad libitum and acclimatized to the facility for seven days prior to starting the experiments. All procedure and handling techniques were in strict accordance with the National Institutes of Health guidelines for the care and use of laboratory animals and approved by the Institutional Animal Care and Use Committee. Animals were randomized into one of two groups (sham or SCI), with the SCI group subjected to a spinal cord contusion injury using the Infinite Horizon (IH) impactor device (Precision Systems & Instrumentation, Lexington, KY) by surgeons blinded to the treatment conditions. Briefly, rats were anesthetized with sodium pentobarbital (Nembutal 40 mg/kg ip, Abbott Laboratories), and the fur overlying the vertebral column was shaved and a dorsal laminectomy was performed at the tenth thoracic vertebra level (T10) to expose the spinal cord. The vertebral column was then stabilized by clamping the rostral T9 and caudal T11 vertebral bodies, so that the spinal cord remained in a horizontal plane. All SCI animals received a 200 kdyn (impact force) spinal cord contusion using the IH impactor device at the T10 level. This injury force results in a moderate to severe contusion injury in female rats in this weight range [14]. Immediately following injury, the muscles and the incision site were sutured in layers, and all animals were injected with 10 cc sterile saline (s.c.) and 30 mg/kg Cefazolin (i.m.). Sham animals received identical surgical procedures but were not subjected to the contusion injury. All animals were then placed on a 37°C heating pad until they recovered fully from the anesthesia. Postoperative care included Cefazolin injections twice a day for one week and the manual expression of bladders twice a day until micturition returned or till the end of the study.

At 42 days following SCI, one set of rats (*n* = 7 SCI, *n* = 4 sham) was deeply anesthetized with 100 mg/kg (ip) sodium pentobarbital (Nembutal; Abbott Laboratories) and transcardially perfused with 100 mL of cold 0.1 M phosphate buffered saline (PBS; pH 7.4) followed by 400 mL of 4% paraformaldehyde in PBS. Animals were then decapitated and the cerebellum was removed and snap-frozen in cold acetone (kept on dry ice) and stored at −80°C until being sectioned on a cryostat for histological analysis. In addition, a different set of injured animals and two sham controls were anesthetized at 24 hr (*n* = 3) or 7 days (*n* = 4) after SCI and decapitated immediately, and the cerebellum was rapidly removed and homogenized in RIPA buffer for western blot analysis of cerebellin-1 (Cbln-1).

### 2.2. Fluorescence Immunohistochemistry

Twenty-micron-thick cryosections of cerebellum from sham and SCI animals were processed using standard immunofluorescence staining procedures routinely conducted in the lab [15, 16]. Based on the Cavalieri principle, every fifth parasagittal section was collected on a series of 10 electrostatically charged glass slides such that adjacent sections mounted on each slide represent cerebellar regions spaced exactly 1 mm apart. Sections mounted onto charged slides were incubated in blocking solution containing PBS, 4% normal goat serum (NGS), and 0.1% Triton X-100. Sections are then incubated overnight at 4°C with a mouse monoclonal antibody to phosphorylated neurofilament (SMI-31, 1 : 1000, Cat. #SMI 31P, Covance) alone or a mixture of SMI-31 and a rabbit polyclonal antibody to synapsin-1 (1 : 1,000, Cat. # ab8, Abcam). The following day, sections were rinsed for 6 × 10 min with PBS and incubated for one hour at room temperature in appropriate rat-absorbed secondary antibodies (1 : 400) conjugated to AlexaFluor 488 (Molecular Probes) or Cy3 (Jackson Labs). Sections were then rinsed for 6 × 10 min in PBS. Antifade solution (ProLong Gold AntiFade solution) and coverslips were placed over the sections. Immunofluorescent staining was visualized and photographed using a Zeiss Axioplan microscope and all analyses focused on cerebellar lobules II and III, which receive ascending axonal input from Clarke's nucleus via the dSCT [11, 12].

### 2.3. Western Blot

Western blot assays were conducted using procedures routinely conducted in the lab [17, 18]. Briefly, cerebellar tissue homogenates were prepared using a Potter-Elvehjem homogenizer containing ice-cold RIPA buffer (50 mM Tris-Cl, 150 mM NaCl, 1% NP-40, 1 mM EDTA, 0.5% sodium deoxycholate, and 0.5% SDS) containing 1% protease inhibitor cocktail (Sigma, Saint Louis, MI), followed by sonication at 100 W for 30 sec. The protein concentration of each sample was measured using the BCA protein assay kit. Samples of total homogenates (10 *μ*g) were then separated by SDS-PAGE using Criterion 4–20% Tris-HCl gels (Bio-Rad, Hercules, CA). The gels were transferred onto PVDF membranes (Millipore) and the membranes were incubated overnight at 4°C with a polyclonal antibody to Cbln-1 (1 : 1000, Cat. #: LS-C119839, LifeSpan Biosciences). The following day, blots were rinsed for 6 × 10 min in TBS buffer containing 0.1% Tween 20 and incubated with rat-absorbed, species appropriate antibodies conjugated to HRP (1 : 4000, Jackson ImmunoResearch). Finally, bots were rinsed for 6 × 10 min followed by incubation in Enhanced Chemiluminescence Plus solution (GE Healthcare) and exposure to chemiluminescent-sensitive film (Hybond CL). The blots were then stripped and reprobed with an antibody to *β*-tubulin (1 : 1000, Cat. # ab6046, abcam), which served as a loading control as well as a normalizing signal. Quantitative densitometric analysis of Cbln-1 bands normalized to *β*-tubulin was conducted using ImageJ software (http://rsb.info.nih.gov/ij/).

### 2.4. Statistical Analysis

Densitometric data from western blot were analyzed by ANOVA and Student-Newman-Keuls post hoc analyses using GraphPad Prism 6 (GraphPad Software, Inc). Differences were considered significant if *p* < 0.05.

## 3. Results

**Figure 1:**
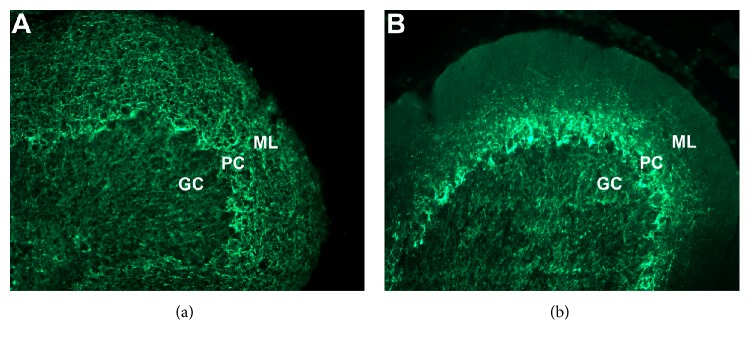
Low-power (20x objective) photomicrographs of SMI-31 axonal staining in lobule III from sham cerebellum (a) and the cerebellum from a 42-day SCI animal (b). Note the prominent decrease of SMI-31 positive immunoreactive profiles in the molecular layer (ML) corresponding to a loss of granule cell parallel fibers. PC: Purkinje cell layer; GC: granule cell region.

The SMI-31 antibody recognizes high molecular weight phosphorylated neurofilaments in intact axons. Cerebellar sections from sham injured animals contained robust SMI-31 staining of axonal process in the granule cell (GC) layer. SMI-31 positive fibers were also observed coursing perpendicular to the lobule surface in the molecular layer (ML), which is consistent with the known projection pattern of the cerebellar parallel fibers (Figure 1(a)). However, SMI-31 immunoreactivity in the cerebellum of 42-day SCI animals revealed a loss of axonal staining in both the GC and the ML of lobule III (Figure 1(b)). The 42-day postinjury time point was chosen due to the fact that, in the absence of a treatment, any behaviorally relevant structural changes have stabilized at this relative chronic time point.

**Figure 2:**
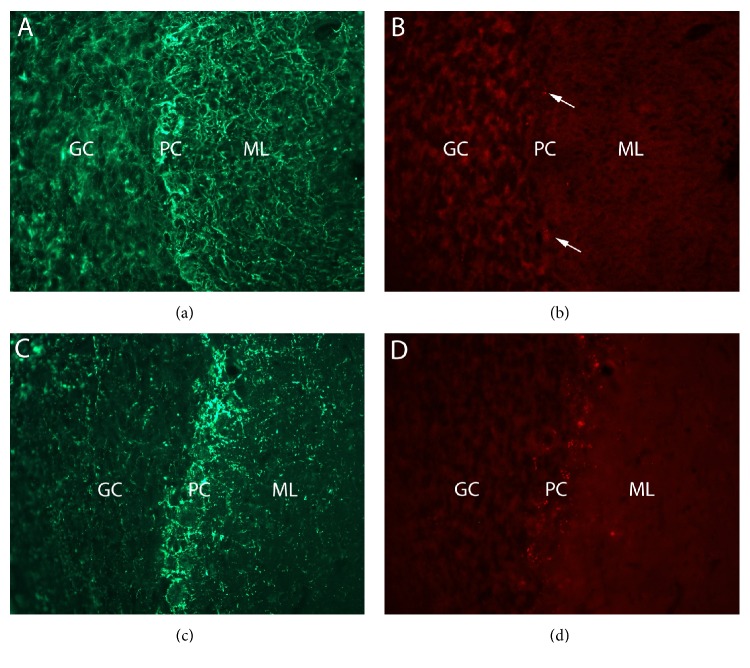
Photomicrographs (40x objective) of SMI-31 ((a) and (c)) and synapsin-1 ((b) and (d)) staining in sham ((a) and (b)) or 42-day SCI cerebellum ((c) and (d)). Note the loss of SMI-31 staining in the molecular layer (ML), as well as the pronounced synapsin-1 staining in the Purkinje cell (PC) layer in the 42-day SCI cerebellum ((c) and (d)).

A loss of parallel fiber input to Purkinje cell (PC) dendrites has been shown to alter the innervation pattern of cerebellar climbing fibers, which originate in the inferior olive and synapse on the PC cell body and proximal dendritic tree. Therefore, additional cerebellar sections were double-labeled with antibodies to SMI-31 and synapsin-1 to visualize SCI-induced changes in Purkinje cell synaptic patterns. As shown in Figure 2(b), a few punctate synapsin-1 immunoreactive profiles were observed in the Purkinje cell bodies in lobule III of sham injured animals (arrows in Figure 2(b)). However, examination of sections from 42-day SCI animals revealed numerous punctate synapsin-1 positive profiles in the lobule III Purkinje cell layer compared to that observed in sham animals (Figure 2(d) compared to Figure 2(b)).

**Figure 3:**
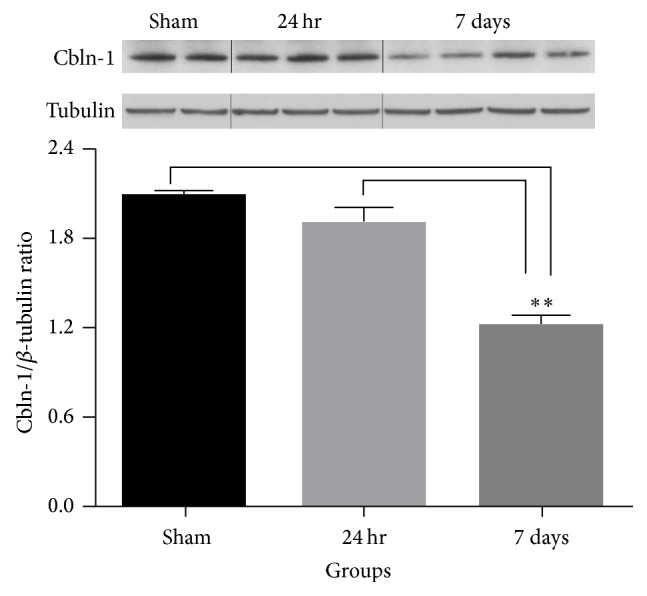
Western blot showing Clbn-1 levels in cerebellum of sham ad SCI animals at 24 hr and 7 days following injury. Densitometric analysis revealed that Cbln-1 levels were significantly reduced (^*∗∗*^ *p* < 0.01) within 7 days after SCI relative to sham and the 24 hr survival time point. Bars represent group means ± SEM.

The loss of parallel fiber staining suggests a disruption of contact with Purkinje cell dendrites. Therefore, we examined cerebellar homogenates from relatively early post-SCI time points for levels of cerebellin-1 (Cbln-1), a cerebellum specific glycoprotein thought to be important for maintaining synaptic integrity of parallel fibers with Purkinje cell dendrites [19, 20]. As shown in Figure 3, densitometric analysis revealed that Cbln-1 levels in cerebellar homogenates from 24 hr SCI animals were no different from those observed in sham animals. However, Cbln-1 levels were found to be significantly reduced within 7 days after SCI relative to sham (46%, *p* < 0.01) and also compared to homogenates obtained from 24 hr post-SCI animals (38%, *p* < 0.01).

## 4. Discussion

The results of this study suggest that a loss of spinocerebellar input due to a contusive injury to the rat thoracic spinal cord alters cerebellar circuitry. This is reflected by a loss of SMI-31 immunoreactive parallel fibers innervating distal segments of Purkinje cell dendrites and an increase in synapsin-1 immunoreactive profiles on cell bodies in the Purkinje cell layer. These structural changes are preceded by a loss of Cbln-1 levels as early as 7 days after injury. We hypothesize that a contusive injury at lower thoracic segments results in a loss of Clarke's nucleus neurons and their axons, which convey ascending nonconscious proprioception feedback to the cerebellum via spinocerebellar input to cerebellar granule cells. As a consequence, there is disruption of granule cell activation and an apparent loss of excitatory parallel fiber influence on the distal Purkinje cell dendrites.

The major output out of cerebellar information to higher motor centers begins with the Purkinje cell, which integrates the activity of two excitatory afferents consisting of the parallel fibers from cerebellar granule cells and climbing fibers originating in the contralateral inferior olive. Parallel fiber axons ascend into the outer regions of the molecular layer where they bifurcate and synapse on distal segments of the Purkinje cell dendritic tree. Climbing fibers ascend into the more superficial territory of the molecular layer and terminate on the Purkinje cell bodies and proximal dendrites. During development, Purkinje cells receive inputs from multiple climbing fibers followed by a pruning phase characterized by a strict innervation pattern where a single climbing fiber innervates a specific Purkinje cell. Several studies have demonstrated that the developmental pattern of multiple climbing fiber inputs to a single Purkinje cell is maintained into adulthood under conditions that disrupt excitatory parallel fiber input to Purkinje cell dendrites.

For example, a loss of granule cells during development results in a loss of parallel fiber input and maintenance of multiple climbing fiber inputs to Purkinje cells [21, 22]. In addition, genetic deletion of Cbln-1, a cerebellum specific glycoprotein expressed in granule cell axons, also leads to a loss of parallel fiber connectivity to Purkinje cell dendrites and expansion of climbing fiber innervation [23]. An almost identical alteration in parallel/climbing fiber organization also occurs in mice lacking the orphan glutamate receptor delta 2 (*Glurδ2*) [24]. Glur*δ*2 is expressed in Purkinje cell dendrites in opposition to Cbln-1-containing parallel fibers, and it has been suggested that Glur*δ*2 and Cbln-1 interact to stabilize parallel fiber-Purkinje cell synaptic integrity [19, 20]. We suggest that the increase in synapsin-1 immunoreactivity on Purkinje cell bodies reflects the presence of additional climbing fiber synaptic contacts that have formed in response to a loss of spinocerebellar input, decreased parallel fiber excitatory activity, or both. Taken together, the reorganizational pattern we observed following SCI is similar to what occurs following developmental loss of granule cells or disruption of Cbln-1/Glur*δ*2 gene expression. In support of this, the relatively early decrease in Cbln-1 levels we observed in the present study is consistent with the developmental literature and Cbln-1 gene deletion studies discussed above.

Overall, the results of this study suggest that cerebellar circuitry undergoes significant alterations in response to injuries of the spinal cord that affect ascending spinocerebellar pathways. This novel observation extends our understanding of the pathophysiological consequences of SCI, which may be critical for developing strategies targeting the recovery of coordinated movement and motor learning.

## References

[1] Rossignol S., Frigon A. (2011). Recovery of locomotion after spinal cord injury: some facts and mechanisms. *Annual Review of Neuroscience*.

[2] Barbeau H., Rossignol S. (1987). Recovery of locomotion after chronic spinalization in the adult cat. *Brain Research*.

[3] Ichiyama R. M., Courtine G., Gerasimenko Y. P. (2008). Step training reinforces specific spinal locomotor circuitry in adult spinal rats. *The Journal of Neuroscience*.

[4] Lovely R. G., Gregor R. J., Roy R. R., Edgerton V. R. (1990). Weight-bearing hindlimb stepping in treadmill-exercised adult spinal cats. *Brain Research*.

[5] Courtine G., Gerasimenko Y., van den Brand R. (2009). Transformation of nonfunctional spinal circuits into functional states after the loss of brain input. *Nature Neuroscience*.

[6] Lavrov I., Gerasimenko Y. P., Ichiyama R. M. (2006). Plasticity of spinal cord reflexes after a complete transection in adult rats: relationship to stepping ability. *Journal of Neurophysiology*.

[7] Edgerton V. R., Courtine G., Gerasimenko Y. P. (2008). Training locomotor networks. *Brain Research Reviews*.

[8] Singh A., Balasubramanian S., Murray M., Lemay M., Houle J. (2011). Role of spared pathways in locomotor recovery after body-weight-supported treadmill training in contused rats. *Journal of Neurotrauma*.

[9] Dobkin B. H., Duncan P. W. (2012). Should body weight-supported treadmill training and robotic-assistive steppers for locomotor training trot back to the starting gate?. *Neurorehabilitation and Neural Repair*.

[10] van den Brand R., Heutschi J., Barraud Q. (2012). Restoring voluntary control of locomotion after paralyzing spinal cord injury. *Science*.

[11] Matsushita M., Gao X. (1997). Projections from the thoracic cord to the cerebellar nuclei in the rat, studied by anterograde axonal tracing. *Journal of Comparative Neurology*.

[12] Hantman A. W., Jessell T. M. (2010). Clarke's column neurons as the focus of a corticospinal corollary circuit. *Nature Neuroscience*.

[13] Palay S., Chan-Palay V. (1974). *Cerebellar Cortex: Cytology and Organization*.

[14] Scheff S. W., Rabchevsky A. G., Fugaccia I., Main J. A., Lumpp J. E. (2003). Experimental modeling of spinal cord injury: characterization of a force-defined injury device. *Journal of Neurotrauma*.

[15] McEwen M. L., Springer J. E. (2005). A mapping study of caspase-3 activation following acute spinal cord contusion in rats. *The Journal of Histochemistry and Cytochemistry*.

[16] Nottingham S. A., Springer J. E. (2003). Temporal and spatial distribution of activated caspase-3 after subdural kainic acid infusions in rat spinal cord. *Journal of Comparative Neurology*.

[17] Chen A., McEwen M. L., Sun S., Ravikumar R., Springer J. E. (2010). Proteomic and phosphoproteomic analyses of the soluble fraction following acute spinal cord contusion in rats. *Journal of Neurotrauma*.

[18] Readnower R. D., Pandya J. D., McEwen M. L., Pauly J. R., Springer J. E., Sullivan P. G. (2011). Post-injury administration of the mitochondrial permeability transition pore inhibitor, NIM811, is neuroprotective and improves cognition after traumatic brain injury in rats. *Journal of Neurotrauma*.

[19] Matsuda K., Miura E., Miyazaki T. (2010). Cbln1 is a ligand for an orphan glutamate receptor *δ*2, a bidirectional synapse organizer. *Science*.

[20] Uemura T., Lee S.-J., Yasumura M. (2010). Trans-synaptic interaction of GluR*δ*2 and neurexin through Cbln1 mediates synapse formation in the cerebellum. *Cell*.

[21] Bravin M., Rossi F., Strata P. (1995). Different climbing fibres innervate separate dendritic regions of the same Purkinje cell in hypogranular cerebellum. *The Journal of Comparative Neurology*.

[22] Crepel F., Mariani J., Delhaye Bouchaud N. (1976). Evidence for a multiple innervation of purkinje cells by climbing fibers in the immature rat cerebellum. *Journal of Neurobiology*.

[23] Hirai H., Pang Z., Bao D. (2005). Cbln1 is essential for synaptic integrity and plasticity in the cerebellum. *Nature Neuroscience*.

[24] Miyazaki T., Yamasaki M., Takeuchi T., Sakimura K., Mishina M., Watanabe M. (2010). Ablation of glutamate receptor glur*δ*2 in adult purkinje cells causes multiple innervation of climbing fibers by inducing aberrant invasion to parallel fiber innervation territory. *Journal of Neuroscience*.

